# Induction of Reactive Intermediates and Autophagy-Related Proteins upon Infection of Macrophages with* Rhodococcus equi*

**DOI:** 10.1155/2017/8135737

**Published:** 2017-11-01

**Authors:** Prashanth Chandramani-Shivalingappa, Mahesh Bhandari, Sarah A. Wiechert, Jessica Gilbertie, Douglas E. Jones, Brett A. Sponseller

**Affiliations:** ^1^Department of Veterinary Microbiology and Preventive Medicine, College of Veterinary Medicine, Iowa State University, Ames, IA 50011, USA; ^2^Division of Pulmonary, Critical Care, and Sleep Medicine, Icahn School of Medicine at Mount Sinai, New York, NY 10029, USA; ^3^Department of Veterinary Pathology, College of Veterinary Medicine, Iowa State University, Ames, IA 50011, USA; ^4^Department of Veterinary Clinical Sciences, College of Veterinary Medicine, Iowa State University, Ames, IA 50011, USA

## Abstract

*Rhodococcus equi (R. equi)* is an intracellular macrophage-tropic pathogen with potential for causing fatal pyogranulomatous pneumonia in foals between 1 and 6 months of age. In this study, we sought to determine whether infection of macrophages with* R. equi* could lead to the induction of autophagy. Murine bone marrow derived macrophages (BMDM) were infected with* R. equi* for various time intervals and analyzed for upregulation of autophagy proteins and accumulation of autophagosomes relative to uninfected controls. Western blot analysis showed a progressive increase in LC3-II and Beclin1 levels in a time-dependent manner. The functional accumulation of autophagosomes detected with monodansylcadaverine further supported the enhanced induction of autophagy in BMDM infected with* R. equi*. In addition, infection of BMDM with* R. equi* induced generation of reactive oxygen species (ROS) in a time-dependent manner. These data are consistent with reports documenting the role of ROS in induction of autophagy and indicate that the infection of macrophages by* R. equi* elicits innate host defense mechanisms.

## 1. Introduction

*Rhodococcus equi* is an aerobic, Gram-positive, nonspore forming coccobacillus which infects foals within the first few weeks of life and can cause severe pyogranulomatous pneumonia between 1 and 6 months of age [1–3].* R. equi* persists and replicates intracellularly in murine and equine macrophages [4, 5]. Upon infection,* R. equi* is phagocytosed and resides in an early phagosome compartment, which fails to undergo later maturation stages thereby resulting in an* R. equi* containing vacuole (RCV). Formation of RCV results in bacterial multiplication and killing of the host macrophage by necrosis with the net effect of* R. equi* escaping macrophage bactericidal activity [6, 7]. A plasmid encoding the virulence-associated protein antigen (VapA) has been demonstrated to be necessary for virulence and replication of* R. equi* in macrophages [8–12].

Macrophages are immune cells involved in eliciting primary responses to pathogens, maintenance of tissue homeostasis, as well as coordination of adaptive immune responses, inflammation, resolution of inflammatory states, and tissue repair [13]. Based on distinct functions and physiological roles, macrophages are activated in response to different innate or adaptive immune signals. Various receptors expressed on the surface of macrophages play an important role in influencing and directing immune responses. Activation of macrophages through Fc*γ*RI and Toll-like receptor (TLR) signaling is required for phagocytosis of pathogens and can also activate NADPH oxidase (NOX2) to trigger the generation of reactive oxygen species (ROS), such as superoxide anion (O_2_^−^) and hydrogen peroxide (H_2_O_2_) which have potential to kill microbes [14, 15]. It has been shown by various studies that mitochondria are the main source of ROS for regulation of autophagy [16, 17] and that NOX2 generated ROS is a key regulator of bacterial autophagy [18].

Autophagy is a highly conserved, degradative process which involves the engulfment of a portion of cell cytoplasm and organelles in double membrane vesicles known as autophagosomes which are themselves targeted for lysosomal degradation [19]. In mammals, regulation of autophagy requires several genes termed “autophagy-related genes” (Atg genes) which collectively have diverse functions [20, 21]. In addition to cytoplasmic material, xenophagy recognizes intracellular pathogens for targeted lysosomal degradation [19, 22]. Xenophagy is an emerging innate defense mechanism against pathogenic agents, including bacteria, viruses, and parasites [22]. Phagocytosis and autophagy act cooperatively as components of the host's innate immune defenses against microbial invasion [18, 23]. Autophagy protein Rubicon is shown to be essential for activation of the NADPH oxidase complex, leading to ROS production upon receptor ligation of TLR-2 or upon microbial infection [24]. Rubicon also negatively regulates the maturation step of autophagy as part of a Beclin1-Vps 34-containing autophagy complex [24, 25]. To extend these studies in the context of macrophage infection with intracellular bacteria, we investigated induction of autophagy in a longitudinal study by detection of the autophagy proteins Beclin1 and LC3-II and indirectly by monitoring production of ROS upon infection of bone marrow derived primary murine macrophages with* R. equi*.

## 2. Methods

### 2.1. Reagents

Brain Heart Infusion (BHI) and Luria-Bertani (LB) agar were purchased from Becton, Dickinson and Company (Sparks, MD). 2-Mercaptoethanol, sodium pyruvate, glucose, MDC (monodansylcadaverine), and mouse anti-*β*-actin monoclonal antibody were obtained from Sigma-Aldrich (St. Louis, MO). Rabbit polyclonal antibody to LC3B and rabbit polyclonal antibody to Beclin1 were purchased from Abcam Inc., (Cambridge, MA) and Santa Cruz Biotechnology (Santa Cruz, CA). IRDye 800 conjugated anti-rabbit antibody was purchased from Rockland Immunologicals (Gilbertsville, PA). CM-H_2_DCFDA, Prolong Gold antifade reagent with DAPI, Alexa Fluor 680 conjugated anti-mouse, and Alexa Fluor 488 anti-rabbit antibodies were supplied by Molecular Probes (Eugene, OR). HEPES, L-Glutamine, penicillin, and streptomycin were purchased from Invitrogen (Carlsbad, CA). Dulbecco's modified Eagle's medium (DMEM) was supplied by Mediatech, Inc. (Manasses, VA). Fetal Bovine Serum was purchased from Valley Biomedical, Inc. (Winchester, VA).

### 2.2. Bacteria

T194* R. equi* strain, a clinical isolate from a pneumonic foal (provided by Dr. Ronald Griffith, Iowa State University), was thawed from −80°C and streaked on an LB agar plate and was grown for 30 hours in 37°C. A single colony was inoculated into 20 ml of Brain Heart Infusion (BHI) media and incubated at 37°C shaker set at 200 rpm to achieve an optical density (O.D) of 0.25. Bacteria were further washed in sterile PBS and resuspended in BHI media.

### 2.3. BMDM Isolation and Cell Culture

Bone marrow cells were harvested from the femur of C3HeB/FeJ mice according to the IACUC approved protocol. Briefly, the bone marrow cells were flushed out using a 25-gauge needle fixed to 10 ml syringe containing complete cell culture medium (CTCM) with Dulbecco's modified Eagle's medium (DMEM) supplemented with 10% Fetal Bovine Serum (FBS), 2 mM glutamine, 0.05 *μ*M 2-mercaptoethanol, 4.5 g/L glucose, 25 mM HEPES, 100 U/ml penicillin, and 100 *μ*g/ml streptomycin. Bone marrow cells were centrifuged at 250 g for 15 min at 4°C and resuspended in a bone marrow medium containing 30% L-cell conditioned medium, 20% FBS, 50% DMEM, 1 mM sodium pyruvate, 2 mM L-glutamine, 100 U/ml penicillin, and 100 ug/ml streptomycin. Approximately 25 × 10^6^ cells were seeded in 15 mm Petri dish and incubated at 37°C in 5% CO2. On day 2 of culture, the media containing nonadherent cells were replaced with a fresh bone marrow medium. On day 6, the adherent bone marrow derived macrophages were harvested and washed in 1x PBS before resuspension in CTCM.

### 2.4. Confocal Microscopy

To evaluate the intracellular replication of* R. equi*, 5 × 10^5^ BMDM were allowed to adhere on coverslips in 24-well plates. Cells were infected with* R. equi* at a multiplicity of infection (moi) of 3 and incubated for 12 h, 24 h, and 48 h at 37°C. Following infection, cells were fixed with 4% PFA in PBS for 15 min, permeabilized with 0.1% Triton-X 100 for 10 min at room temperature (RT), and blocked with a buffer (10% goat serum, 0.4% bovine serum albumin in 1x PBS) for 30 mins. To label intracellular bacteria, cells were incubated with rabbit polyclonal* R. equi* (1 : 3000) as primary antibodies for an hour [26]. The coverslips were washed thrice with 1x PBS for 5 min and incubated with goat anti-rabbit Alexa 488 (1 : 3000) as secondary antibodies for 1 h. Coverslips were further washed three times with 1x PBS and mounted on glass slides using ProLong with DAPI (Molecular Probes, Invitrogen). Confocal microscopy was performed either using an Olympus IX-61 microscope equipped with red, green, and blue filter sets with a cooled CCD camera or by an inverted Olympus Fluoview™ 1000 (Minneapolis, MN) laser-scanning microscope.

### 2.5. ROS Assay

Approximately 50,000 cells in complete cell culture medium (CTCM) were seeded per well in a 96-well plate. After infection, cells were incubated with 10 *μ*M CM-H_2_DCFDA dye (Invitrogen) in the dark at 37°C for 30 mins. Cells were washed with Hank's balanced salt solution (HBSS) with calcium and magnesium. Fluorescence was measured using a Fluostar Omega (BMG Biotech) fluorimeter with setup excitation at 485 nm and emission at 540 nm.

### 2.6. MDC Assay

The MDC (monodansylcadaverine) assay to label autophagosomes has been described previously [27]. After infection, macrophages were incubated with 0.05 mM MDC in a serum-free RPMI medium at 37°C for 30 mins. Later, macrophages were harvested and lysed in 10 mM Tris-HCl, pH 7.4 containing 1% Triton-X 100. Accumulation of MDC in autophagy vacuoles was measured using a Fluostar Omega plate reader (BMG LABTECH Inc., NC, USA) with excitation and emission wavelength set at 355 nm and 540 nm. Further, the number of cells in each well was normalized by the addition of 0.2 *μ*M ethidium bromide and the DNA fluorescence was measured with excitation at 485 nm and emission at 670 nm. Incorporation of MDC was expressed as activity.

### 2.7. Western Blotting

After infection, cells were harvested and lysates were prepared in RIPA lysis buffer (25 mM Tris-HCl (pH 7.6), 150 mM NaCl, 1% NP-40, 1% sodium deoxycholate, and 0.1% SDS) containing 1x protease inhibitor cocktail (Pierce). Protein quantification was performed using a Bradford protein assay kit. 50 *μ*g of protein were separated on a 10% or 15% SDS-PAGE. In addition to blocking nonspecific sites (1 h) by the use of LICOR blocking buffer, membranes were incubated overnight with rabbit polyclonal to Beclin1 (1 : 500), rabbit polyclonal to LC3B (1 : 4000), and mouse monoclonal *β*-actin (1 : 5000) as primary antibodies followed by an hour incubation with anti-rabbit IR dye-800 (1 : 5000) and anti-mouse Alexa Flour 680 (1 : 15,000) as secondary antibodies. Western blot images were captured using the Odyssey IR Imaging system (LICOR) and data were analyzed with Odyssey software 2.0.

### 2.8. Data Analysis

Data were analyzed by using Prism 4.0 software (GraphPad Prism, San Diego, CA). Results represent mean ± SEM. Statistical analysis was performed with one-way ANOVA followed by Tukey's post hoc test (GraphPad Prism software) in order to compare between treatment groups. Results were considered significantly different if *p* < 0.05.

## 3. Results

### 3.1. *R. equi* Growth in Bone Marrow Derived Macrophages

**Figure 1:**
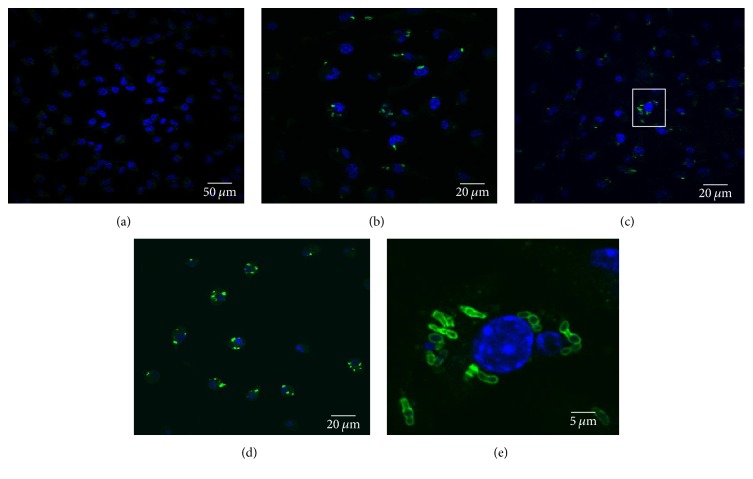
Internalization of* R. equi* in BMDM. Representative confocal immunofluorescence images showing intracellular localization of* R. equi* in BMDM at various time points after infection: (a) uninfected, (b) 12 h, (c) 24 h, (d) 48 h, and (e) 240x magnification of the inset from image (c).* R. equi* are labeled green with rabbit polyclonal* R. equi* antibodies and the nuclei are labeled blue with DAPI. Increasing bacterial load is observed in infected BMDM at later times after infection. Pictures are representative of results obtained from three independent experiments.

First, to investigate the intracellular growth of* R. equi*, harvested and cultured BMDM were grown on coverslips and were infected with* R. equi* (moi3). After 2 hrs, extracellular bacteria were killed using gentamycin sulfate (10 ug/ml). Intracellular bacterial infection was allowed for 12 h, 24 h, and 48 h. BMDM were immunostained with anti-*R. equi* antibody and fluorescence microscopic analysis was performed. In comparison to uninfected BMDM (Figure 1(a)), we observed intracellular* R. equi* as green fluorescence puncta bodies at 12 h (Figure 1(b)). Moreover, significant increase in* R. equi* was observed in 24 h and 48 h (Figures 1(c) and 1(d)). Further, higher magnification of intracellular* R. equi* staining at 24 h revealed that* R. equi*, in the form of clusters and aggregates, localized in the perinuclear region (Figure 1(e)). This finding verifies the previous study by Hondalus and Mosser [5] showing that intracellular* R. equi* replicates in clusters.

### 3.2. ROS Increase in Bone Marrow Derived Macrophages Infected with* R. equi*

Increase in ROS levels in macrophages in response to bacterial invasion is an important bactericidal mechanism. Therefore, it was important to observe whether* R. equi* stimulated the increase in ROS levels in BMDM.

**Figure 2:**
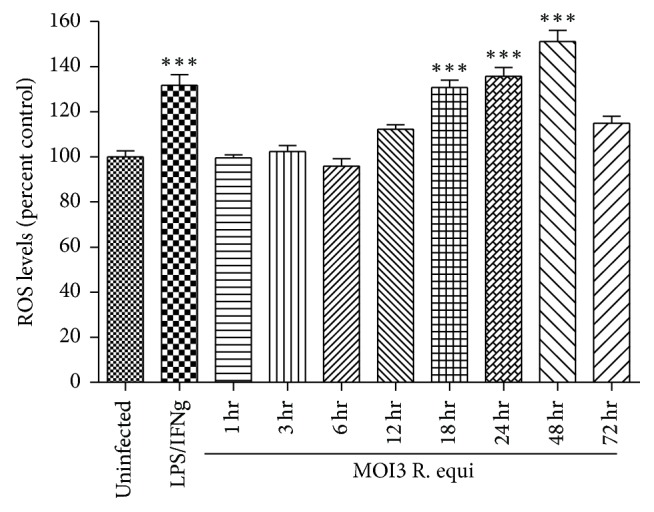
*R. equi* infection of BMDM led to an increase in ROS. BMDM were uninfected or infected with* R. equi* (moi3) for 1 h, 3 h, 6 h, 18 h, 24 h, 48 h, and 72 h. Stimulation of BMDM with a combination of 10 *μ*g/ml LPS and 25 ng/ml IFN*γ* (24 h) is used as a positive control.* R. equi* infection led to an increase in ROS as measured using a ROS indicator, CM-H2DCFDA (30 min). Data represent mean ± SEM of 6 independent measurements. ^*∗∗∗*^*p* < 0.001.

After infection with* R. equi* (moi3) for 12 h, 24 h, 48 h, and 72 h, BMDM were incubated with an ROS indicator CMH_2_DCFDA as described in the Methods. In comparison to uninfected cells, no significant increase in intracellular ROS levels was observed in 1 hr, 3 hr, or 6 hr. Later time point showed significant ROS increase in a time-dependent manner at 18 h (*p* < 0.001, 1.37-fold), 24 h (*p* < 0.001, 1.38-fold), and 48 h (*p* < 0.001, 1.5-fold). We observed a 1.5-fold decrease in intracellular ROS levels at 72 h as compared to 48 h (Figure 2). Stimulation of BMDM with 10 ug/ml LPS and 25 ng/ml IFN g for 24 h was considered as positive control [28, 29] and showed a significant increase (*p* < 0.001; app. 1.3-fold) in ROS levels. The results of these experiments suggest that* R. equi* infection elicited the increase in ROS production in the later time points that would result in cell death by necrosis causing failure to sustain ROS at 72 h.

### 3.3. Induction of Autophagy in Bone Marrow Derived Macrophages Infected with* R. equi*

**Figure 3:**
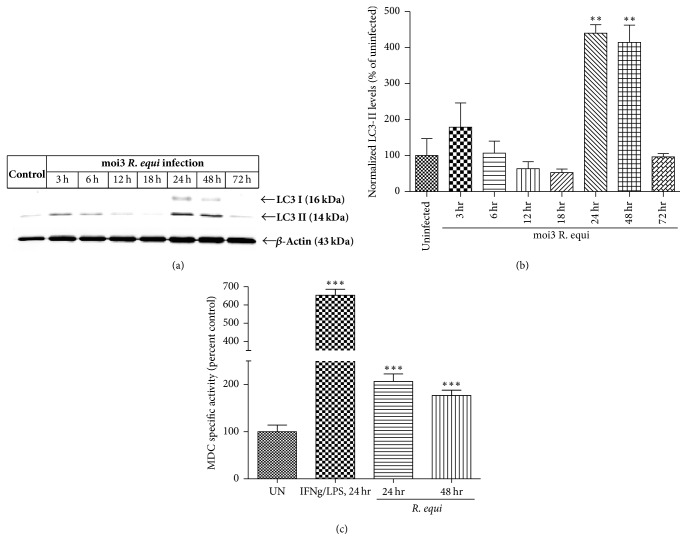
*R. equi* infection of BMDM leads to autophagy induction. (a) Western blotting analysis showing the expression of LC3-II levels in the whole cell protein lysates of BMDM infected with* R. equi* (moi3). LC3-II expression was observed at 24 h and 48 h. *β*-Actin immunoblot represents equal protein loading. Densitometry analysis was used to quantify the density of LC3-II bands. The ratio of LC3-II bands to that of *β*-actin was determined and the data are expressed as percent of control. Data represent mean ± SEM, ^*∗∗*^*p* < 0.01 compared with untreated group. (b) Following infection of BMDM with* R. equi* (moi3), cells were stained with MDC and the fluorescence was measured by a plate reader. A marked increase in MDC staining was evidenced in BMDM at 24 h and 48 h of* R. equi* infection. The results are expressed as mean ± SEM of 6 independent measurements. ^*∗∗*^*p* < 0.001; ^*∗∗∗*^*p* < 0.001.

Recently, the role of autophagy has been implicated as an important host immune response against microbial infections [22]. In this study we aimed to evaluate the regulation of autophagy by* R. equi*. BMDM were infected with* R. equi* (moi3) for 3 h, 6 h, 12 h, 18 h, 24 h, 48 h, and 72 h. Next, we isolated total protein lysates and subjected the lysates to western blotting detection of the autophagy marker, LC3-II a structural component of autophagosomes [30]. In comparison to uninfected control, significant increase in LC3-II levels was observed at 24 h (*p* < 0.01) and 48 h (*p* < 0.01). No significant increase in LC3-II expression was observed in earlier infection time points, including 3 h, 6 h, 12 h, and 18 h. Moreover, at 72 h infection, there was an approximately 4.2-fold decline in LC3-II levels in comparison to 24 h and 48 h infection, suggesting that 72 h infection might lead to necrosis (Figure 3(a)). We further extended this study to observe the formation of autophagy vacuoles. BMDM were infected with* R. equi* (moi3) for 24 h and 48 h. Later, BMDM were incubated with a fluorescent compound called monodansylcadaverine (MDC) to label autophagy vacuoles. Fluorescence measurement showed a significant increase in accumulation in MDC stained autophagy vacuoles at 24 h (*p* < 0.001) and 48 h (*p* < 0.001) infection time points (Figure 3(b)) indicating the induction of autophagy. Stimulation of BMDM with 10 ug/ml LPS and 25 ng/ml IFN g for 24 h was considered as positive control showing a significant increase (*p* < 0.001; app. 6.5-fold) in autophagy vacuoles.

**Figure 4:**
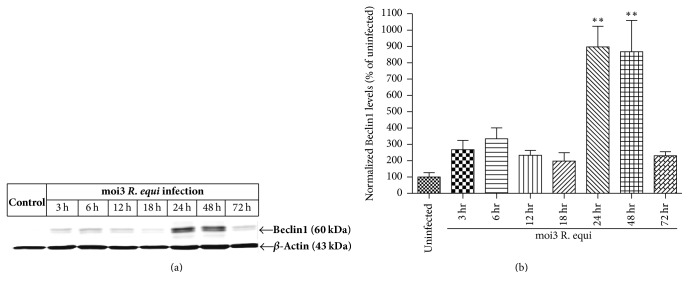
Western blotting analysis showing the expression of Beclin1 levels in the whole cell protein lysates of BMDM infected with* R. equi* (moi3). (a) Beclin1 expression was observed at 24 h and 48 h. Uninfected BMDM were considered as control. *β*-Actin immunoblot represents equal protein loading. (b) Densitometry analysis was used to quantify the density of Beclin1 bands. The ratio of Beclin1 bands to that of *β*-actin was determined and the data are expressed as percent of control. Data represent mean ± SEM, ^*∗∗*^*p* < 0.01.

Next we wanted to clarify if initiation of autophagy has occurred. The whole cell lysates from* R. equi* infected BMDM at various time points (3 h, 6 h, 12 h, 18 h, 24 h, 48 h, and 72 h) were again subjected to immunoblotting of Beclin1 which is a key regulator in the initiation of autophagy and autophagosome formation through class III PI3K pathway [31]. As shown in Figure 4, we observed the expression levels of Beclin1 at later infection time points, 24 h (*p* < 0.01) and 48 h (*p* < 0.01), in comparison to an uninfected control and earlier infection time points (3 h, 6 h, 12 h, and 18 h). Expression level of Beclin1 declined significantly (app. 4.35-fold) at 72 h. Taken together, these results indicate that* R. equi* infection resulted in autophagy and that the autophagy process is a normal host response and further implicates the importance of class III PI3K pathway in bacterial autophagy.

## 4. Discussion

*R. equi* remains a great challenge to the health of young foals and immunocompromised adult horses. It is also a pathogen of immunocompromised humans [30–32].* R. equi* is particularly well recognized as a major cause of pneumonia in foals [33]. The incidence of* R. equi* pneumonia appears to be increasing, possibly because of intensified management of equine breeding farms and climate change. This disease continues to be a major challenge for clinicians and the equine industry in terms of epidemiological pattern and therapeutic control due to its complex host-pathogen interaction [8, 33]. The objective of this study was to correlate the induction of LC3-associated phagocytosis with production of ROS in order to gain insight into the macrophage response upon infection with* R. equi*.

*R. equi* is an intracellular macrophage-tropic Gram-positive coccobacillus which possesses the capability to survive and multiply in murine and equine macrophages [5, 34]. Following phagocytosis,* R. equi* interferes with phagosomal maturation and suppresses acidification of the phagosome to prevent the formation of phagolysosomes [6, 35]. In addition to the interference in phagosomal maturation, our data suggest that infection with* R. equi* induces autophagy, a host response broadly characterized as being protective. Murine BMDM were infected with* R. equi* at an moi of 3 and the regulation of autophagy was analyzed at specific time points after infection. Detection of autophagy-related proteins, LC3-II and Beclin1, by western blot occurred as early as 3 h with significantly higher amounts of these proteins detected at 24 h and 48 h suggesting that the autophagy machinery could play a role in innate host defense. This study was supported by a progressive accumulation of autophagosomes as detected using a MDC detection assay.

Autophagy is a cytoprotective mechanism in which its own cytoplasmic contents are engulfed into autophagosomes and destined for lysosomal degradation. The autophagy mechanism is well studied in various disease states such as cancer, neurodegenerative diseases, aging and immunity, and inflammation [22]. In order to survive and replicate within host cells, some microorganisms have evolved a mechanism to escape degradation by the autophagy machinery [36]. A recently intriguing concept of noncanonical autophagy is characterized by the formation of autophagosomes without the necessity of Atg proteins that are recruited upon a double membrane vacuole containing bacteria [19, 37]. LC3-associated phagocytosis involves the formation of a single membrane autophagosome decorated with LC3 within professional phagocytes. This process is independent of the ULK1 complex [38] but requires NADPH oxidase activity with production of superoxide and other reactive intermediates within the vacuole to exert a bactericidal effect [39]. It has been shown that mycobacterial species, including* Mycobacterium tuberculosis (M. tuberculosis)* and* Mycobacterium marinum (M. marinum)*, survive in macrophages by preventing both phagolysosome formation [40] and recruitment of LC3 to its phagosome [41]. Since* R. equi* and* Mycobacterium* spp. belong to the mycolata taxon and* R. equi* has been shown to inhibit phagolysosomal fusion [6], it is reasonable to speculate that infection with* R. equi* could induce the same innate host response as infection with* M. marinum*. Our data are consistent with the notion that induction of autophagy could contribute to the ability of host cells to control infection by intracellular* R. equi*. A better understanding of this host-pathogen relationship is desirable as there is intense interest in gaining insight into potential therapeutic approaches involving regulation of autophagy.

Depending on the interaction with class III phosphoinositide 3-kinase, Beclin1 plays an important role in localizing autophagy proteins to preautophagosomal structures. In this study, we observed significant levels of Beclin1 at 24 h and 48 h. At this point, it appears that Beclin1 plays an important role in the macrophage's response to infection with* R. equi*, but further studies are needed to explore the role of Beclin1 in regulating* R. equi* induced autophagy. It has been shown that* M. tuberculosis* can escape the phagosome into the cytosol leading to host necrotic cell death via an ESX-1 dependent mechanism. In the study reported here, microscopic necrotic cell death of murine BMDM was observed at 24 h after infection with an moi of 3* R. equi* (results not shown), which is consistent with the documented observation that* R. equi* contains an esx gene cluster encoding ESAT-6 and CFP-10 which are important in virulence and pathogenesis, including necrotic cell death and bacterial release [42, 43]. It is unclear what specific role the autophagy machinery plays upon recognition of* R. equi* in the cytosol and whether it confers a protective effect, possibly delaying host cell necrosis.

Another protective mechanism that appears to be shared by* M. tuberculosis* and* R. equi* is mediated by the histone-like protein,* lsr2* gene, and the* R. equi* homologue, which apparently confer resistance against macrophage produced reactive intermediates of oxygen, such as superoxide [44]. However, despite mechanisms of pathogen resistance to ROS, generation of ROS also plays an important mechanistic role in the induction of autophagy by upregulating the NADPH oxidase complex [25]. In this study, when murine BMDM were infected with* R. equi* at an moi of 3, we observed a time-dependent production of ROS from 18 h until 48 h after infection. It is unclear what the precise mechanism is whereby infection with* R. equi* leads to enhanced production of ROS and induction of autophagy in macrophages. Although these two events could be independent, recent studies have shown that the RUN domain of Beclin1 interacts with the cysteine-rich Rubicon autophagy protein which, in turn, acts in response to microbial infection as a positive regulator of NADPH oxidase [25]. After microbial infection or ligation of TLR2, Rubicon interacts with the p22phox subunit of the NADPH oxidase complex, accelerating its phagosomal trafficking to induce a burst of ROS and inflammatory cytokines. Rubicon's actions in autophagy and phagocytosis are shown to be functionally and genetically separable while the outcome of both of these functions depends on the environmental stimuli [24, 25, 38]. However, it is plausible that both autophagy and phagocytosis play important roles in macrophage resistance to infection with* R. equi*, as Bonilla et al. (2013) showed that autophagy modulates phagocytosis by regulation of scavenger receptors and is required for in vivo eradication of* Mycobacterium* [45].

The data obtained in the present study provide new insights into the macrophage response upon infection with* R. equi*, particularly with respect to induction of autophagy and production of reactive intermediates. Similar kinetics in the upregulation of autophagy protein levels of LC3-II and Beclin1 indicated induction of autophagy upon infection of murine BMDM with* R. equi*. Accumulation of autophagosomes concomitant with an increase in autophagy proteins further supported enhanced induction of autophagy upon infection. ROS generation by murine BMDM is consistent with the necessary signaling role of superoxide in the upregulation of LC3-associated phagocytosis but could also be due to other responses to infection by* R. equi*. Further studies are needed to characterize the signaling pathways involved in regulation of autophagy in* R. equi* infected macrophages and whether modulation of autophagy can be used to mitigate* R. equi* infection in macrophages.
